# Antigenicity of a Bacterially Expressed Triple Chimeric Antigen of *Plasmodium falciparum* AARP, MSP-3_11_ and MSP-1_19_: PfAMSP-Fu_35_

**DOI:** 10.1371/journal.pone.0165720

**Published:** 2016-10-31

**Authors:** Aakanksha Kalra, Jyotheeswara Reddy Edula, Puneet Kumar Gupta, Alok Kumar Pandey, Virander S. Chauhan

**Affiliations:** Malaria Research Group, International Centre for Genetic Engineering and Biotechnology, Aruna Asaf Ali Marg, New Delhi, India; Ehime Daigaku, JAPAN

## Abstract

Development of fusion chimeras as potential vaccine candidates is considered as an attractive strategy to generate effective immune responses to more than one antigen using a single construct. Here, we described the design, production, purification and antigenicity of a fusion chimera (PfAMSP-Fu_35_), comprised of immunologically relevant regions of three vaccine target malaria antigens, PfAARP, PfMSP-3 and PfMSP-1. The recombinant PfAMSP-Fu_35_ is expressed as a soluble protein and purified to homogeneity with ease at a yield of ~ 7 mg L^-1^. Conformational integrity of the C-terminal fragment of PfMSP-1, PfMSP-1_19_ was retained in the fusion chimera as shown by ELISA with conformation sensitive monoclonal antibodies. High titre antibodies were raised to the fusion protein and to all the three individual components in mice and rabbits upon immunization with fusion chimera in two different adjuvant formulations. The sera against PfAMSP-Fu_35_ recognized native parasite proteins corresponding to the three components of the fusion chimera. As shown by invasion inhibition assay and antibody mediated cellular inhibition assay, antibodies purified from the PfAMSP-Fu_35_ immunized serum successfully and efficiently inhibited parasite invasion in *P*. *falciparum* 3D7 *in vitro* both directly and in monocyte dependent manner. However, the invasion inhibitory activity of anti-AMSP-Fu_35_ antibody is not significantly enhanced as expected as compared to a previously described two component fusion chimera, MSP-Fu_24_. Therefore, it may not be of much merit to consider AMSP-Fu_35_ as a vaccine candidate for preclinical development.

## Introduction

There have been increasing efforts in prevention and treatment strategies to control morbidity and mortality caused by malaria. These strategies have cumulatively resulted in ~ 18% and 48% reduction in malaria mortality rates and malaria cases respectively between 2015 and 2000 [1]. However, an estimated 214 million people were still at risk and about 438,000 have lost their lives in 2015 due to increasing resistance of vectors to insecticides and parasites to drug therapies [2–4]. This gradually increasing resistance and these startling numbers have been a strong reminder that an effective vaccine is needed to combat malaria.

Vaccine development efforts to malaria have been targeted to all stages of the parasite’s life cycle *viz* sexual [5,6], pre-erythrocytic [7–9] and erythrocytic [10–12] utilising multiple approaches. These mainly include use of naked DNA, viral vectors to deliver relevant DNA sequences, prime/boost DNA vaccines that include recombinant DNA, viruses and proteins, vaccines based on whole sporozoite, synthetic peptides and recombinant protein(s) with adjuvant [13]. In principle DNA based vaccines are most attractive in that they are simple to design with a possibility of including multiple B and T cell epitopes from different antigens, easy to produce and do not require strong adjuvants to generate significant immune response particularly cellular responses. However, many multiple epitope based DNA vaccines did not live up to expectations and currently there is no DNA vaccine that has been commercialized. A naked DNA based vaccine comprising of PfCSP failed to induce any significant immune responses in human trials [13]. Heterologous prime/boost vaccine strategy is another attractive approach being used in developing vaccines against malaria. For example, delivery of ME-TRAP (multiepitope string- thrombospondin-related adhesion protein) by priming with ChAd63 (chimpanzee adenovirus 63) followed by a booster with modified vaccinia virus (MVA) has induced significantly high cellular responses in malaria naïve and malaria exposed individuals [14]. This prime/boost strategy is being explored for vaccine development in other disease conditions including cancer and HIV [15]. On the other hand, with the seemingly inherent limitations like design of constructs involving multiple epitopes from different antigens or large scale production, recombinant protein(s) based vaccines have shown more promise in malaria. RTS,S, a pre-erythrocytic stage vaccine based on recombinant protein technology, is the most advanced malaria vaccine which has successfully completed Phase III clinical trials and received a positive regulatory assessment by WHO [16]. This has raised hopes for more effective malaria vaccines based on recombinant protein platforms to be developed in future.

Since the clinical manifestations of the disease are caused by blood stage and also most of the parasite’s life cycle in humans occurs in this stage, vaccines targeting blood stage have also been considered essential for effective disease control. A plethora of proteins from blood stage of parasite have been analyzed for their potential as vaccine candidates and this number has risen rapidly in the post genomic era. Merozoite surface proteins (MSPs) belong to an important family of surface proteins including prominent vaccine targets like PfMSP-1 and PfMSP-3. PfAMA-1 is one of the micronemal proteins, antibodies to which inhibit parasite invasion alone or in combination both *in vitro* and *in vivo* [17] but high degree of sequence polymorphism has prevented its successful use as a vaccine [18]. Two micronemal proteins, PfRipr and CyRPA have been shown to form a complex with an important rhoptry protein (PfRH5) thereby securing the complex to parasite surface and enabling binding of PfRH5 to its receptor and facilitating erythrocyte invasion [19]. PfRH5 with its high invasion inhibitory efficiency *in vitro* with strain transcending effects is being pursued as a vaccine candidate antigen [20]. Likewise in our ongoing work to identify vaccine target antigens, we identified a novel protein, Apical Asparagine Rich Protein (PfAARP), localized in rhoptry neck, antibodies to which (particularly its N-terminal fragment or PfAARP ectodomain) efficiently inhibited parasite invasion *in vitro* [21].

Various studies with several antigens have suggested that the quality, level and breadth of antibody response are critical components of protective immunity and therefore a combination of antigens is likely to be more efficacious than a single antigen as a malaria vaccine [22]. Fusion constructs containing more than one malaria protein have been developed which have the additional advantage of expression and purification of a single polypeptide compared to a cocktail of antigens. In fusion constructs, the individual components may be from same stage [23,24] or different stages [25,26] of development of the parasite life cycle. We have recently described a fusion chimera, PfMSP-Fu_24_, in which two epitopes, one from PfMSP-3 and the other from PfMSP-1, were fused together and produced as a single protein [27]. We found that functionally active high titre antibodies were raised to both the epitopes without any antigenic interference [27]. Our recent work with PfAARP as a potential antigen for a cocktail malaria vaccine, and successful production and characterization of the fusion chimera, PfMSP-Fu_24_, have led to the design of a triple fusion construct comprising of the relevant epitope of PfAARP and the bivalent fusion chimera. Thus, the triple construct PfAMSPFu_35_ (henceforth AMSP-Fu_35_ unless stated) comprises of the N-terminal fragment of PfAARP (20–107 amino acids) [21], 9kDa (PfMSP-3_11_) region of PfMSP-3 [28] followed by 19kDa (PfMSP-1_19_) region of PfMSP-1 [29] at the C-terminus. This fragment of PfAARP binds erythrocytes in neuraminidase and trypsin sensitive manner [21]. It has also been recognized by sera from malaria affected individuals residing in *P*. *falciparum* endemic region pointing to its role in naturally acquired immunity. Our recent studies have shown that in addition to a high titre long lasting humoral response, PfAARP also induces an effective cellular response and that PfAARP sequence is comprised of three linear B cell and one T cell epitopes [30]. Also, antibodies to PfAARP ectodomain have been shown to block parasite invasion *in vitro* as well as provide synergistic effects in inhibition of merozoite invasion when used in combination with antibodies to a number of parasite antigens including PfRH1, PfF2, PfRH2, PfRH5, and PfTRAMP [21,31]. Further, anti-PfAARP antibodies when used in combination with antibodies to PfRH2 and PfF2, showed inhibition of parasite invasion in strain transcending manner [31]. Lack of strain transcending effects has been a major problem for crucial vaccine target antigens like PfAMA-1 [32].

The fragments PfMSP-3_11_ of PfMSP-3 and PfMSP-1_19_ of PfMSP-1 were used in this fusion construct because of their following advantages: i) PfMSP-3_11_, the 70 amino acid fragment of PfMSP-3 is known to possess T helper epitopes to which high levels of cytophilic antibodies have been directed [28]; ii) In combination with GLURP, N-terminal region of PfMSP-3 induced high titre antibodies and memory B cells [23]; iii) highly conserved PfMSP-1_19_ fragment neutralized merozoites, inhibited parasite invasion *in vitro* and reduced parasitemia and disease severity *in vivo* [29]; iv) fusion of PfMSP-3_11_ to PfMSP-1_19_ raised the titre of inhibitory antibodies specific to PfMSP-1_19_ [27,33]; v) in combinations with other blood stage antigens both the fragments have been involved in clinical trials [34].

AMSP-Fu_35_ was expressed in *E*. *coli* as a soluble protein and was purified to homogeneity with ease. We found that AMSP-Fu_35_, formulated with two different adjuvants, was highly immunogenic in small animals. There was no antigenic competition and a high antibody response was observed to the three individual components. Anti-fusion chimera antibodies efficiently inhibited parasite invasion *in vitro* both directly and in a monocyte dependent manner.

## Materials and Methods

### Bacterial strains, antibodies and matrices

*E*. *coli* strain BLR (DE3) (Novagen, EMD Millipore, USA) was used for expression of AMSP-Fu_35_. Streamline Chelating and Q-Sepharose FF used for purification of recombinant protein were obtained from GE Healthcare, Sweden. C-8 RP-HPLC column was purchased from Discovery Supelco. Complete Freund’s Adjuvant/Incomplete Freund’s Adjuvant (Sigma Aldrich) and Alhydrogel^(R)^ (Brenntag, Denmark) adjuvants have been used for immunization of small animals. HRP conjugated anti-mice and anti-rabbit secondary antibodies were purchased from Sigma.

### Construction of plasmid expressing AMSP-Fu_35_

The fusion construct, AMSP-Fu_35_, is comprised of fragments from three parasite proteins. Sequentially, the construct is comprised of 10kDa region of PfAARP (Gene ID: PF3D7_0423400) at the N terminus which is followed by a 9kDa fragment of PfMSP-3 (Gene ID: PF3D7_1035400) with the 19kDa fragment of PfMSP-1 (Gene ID: PF3D7_0930300) at the C terminus. A synthetic gene was constructed based on this fusion chimera by back translation to the nucleotide sequence built on the *E*. *coli* codon frequency table (based on most frequently used codons) as described at http://www.kazusa.or.jp/codon. This gene was commercially synthesized and sub cloned in pET24b (+) (GeneArt, Life Technologies) with a C-terminal His tag between Nde 1 and Xho 1 restriction sites.

### Expression, purification and characterization of the recombinant protein

The recombinant plasmid containing AMSP-Fu_35_ gene was transformed in *E*. *coli* BLR (DE3) cells and the transformed cells were grown in APS-Select media containing kanamycin (50μg/ml) at 37°C to reach an optical density of 0.6 to 0.8 at 600nm (OD_600_). The expression of the recombinant protein was induced by 1mM isopropyl-β-D-thiogalactopyranoside (IPTG) for 4hrs and expression was analyzed in un-induced and induced samples by SDS-PAGE. The expression levels were checked in soluble and insoluble fractions of *E*. *coli* cells after disruption by SDS-PAGE and western blot analysis.

For purification of the recombinant protein, the cell pellet from 6L shake flask culture was washed with phosphate buffer saline (PBS), pH7.4 for the removal of toxic components of the spent media and to maintain osmolarity. This was followed by re-suspension and homogenization of the cell pellet in lysis buffer (20mM Tris pH 8.0, 500mM NaCl, 5mM Benzamidine-HCl, 10mM Imidazole & 100μg/ml lysozyme). The homogenized cells were further lysed by sonication on ice with a 9sec pulse on/off. The lysed culture was centrifuged and resulting clear supernatant was loaded on to equilibrated Ni^2+^ charged streamline chelating resin (equilibration buffer: 20mM Tris pH 8.0, 500mM NaCl). The resin was subsequently washed with five column volumes each of wash buffer 1 (20mM Tris, 500mM NaCl & 10mM Imidazole pH 8.0), wash buffer 2 (20mM TrisHCl & 500mM NaCl pH 8.0) and wash buffer 3 (20mM TrisHCl, 10mM NaCl & 40mM Imidazole pH8.0). Bound protein was eluted with a step gradient of imidazole (40mM to 500mM) in 20mM Tris and 10mM NaCl pH 8.0. The protein containing fractions were loaded on to equilibrated Q-Sepharose resin (equilibration buffer = 20mM TrisHCl & 10mM NaCl) for further purification. Bound protein was eluted with a step gradient of NaCl (10mM to 500mM) in 20mM TrisHCl pH 8.0; the eluates containing AMSP-Fu_35_ were pooled and quantified by bicinchoninic acid assay (BCA).

Identity of the recombinant protein was analysed by immunoblotting with monoclonal anti-His antibody as per standard protocols. Purity of the protein was assessed by SDS-PAGE under reducing and non-reducing conditions and by reverse phase chromatography. Endotoxin content of the protein was estimated by Limulus amoebocyte lysate (LAL) gel clot assay (Charles River Endosafe, USA) as per standard protocol. Conformational integrity of PfMSP-1_19_ fragment in recombinant AMSP-Fu_35_ was analyzed by ELISA and immunoblotting using conformation sensitive monoclonal antibodies (MAbs) 2E10 and 1H4 [35] as described in further sections. The presence of free thiol groups in the recombinant protein was detected by Ellman’s test wherein the test sample was compared to a dilution series (0mM to 1.5mM) of the standard, cysteine hydrochloride. Both the standard and the test samples were incubated with Ellman’s reagent (5’-dithio-bis-3-nitro benzoic acid) at room temperature for 15min and absorbance was measured at 412nm.

Constituent protein fragments of AMSP-Fu_35_, namely PfAARP, PfMSP-3_11_ and PfMSP-1_19_ were prepared by recombinant methods which were purified and characterized as described earlier [21,33].

### Immunization of mice and rabbits with recombinant antigens formulated with Freund’s adjuvant and Alhydrogel

The parent strains of BALB/c mice and New Zealand White (NZW) female rabbits were obtained from the Jackson Laboratory and National Institute of Nutrition, Hyderabad respectively and were further bred by the animal housing facility of ICGEB under pathogen-free conditions as recommended by the Guide for the Care and Use of Laboratory Animals (ICGEB, India). ICGEB is licensed to conduct animal studies for research purposes under the registration number 18/1999/CPCSEA (dated 10/1/99). All the experimental protocols were approved by the ICGEB Institutional Animal Care and Use Committee (IAEC: MAL).

Groups of six female BALB/c mice (6 to 8 weeks old) were immunized subcutaneously with 25μg of AMSP-Fu_35_ formulated with Complete Freund’s Adjuvant/Incomplete Freund’s Adjuvant and Alhydrogel^(R)^. In parallel, groups of five mice were immunized separately with 25μg of all the three component proteins; PfAARP, PfMSP-1_19_, or PfMSP-3_11_ formulated with the two adjuvants. The animals were given booster doses on days 28 and 56 post-immunization and were bled on day 0, 14, 42, 70. In groups of two, 3–4 months old NZW rabbits were immunized subcutaneously with 100μg of AMSP-Fu_35_ formulated with both adjuvants followed by 2 booster immunizations on days 28 and 56 and were bled 14 days after the last immunization (Day 70).

### Immunoblotting

All the immunoblotting experiments were performed using Nitrocellulose membrane (NC) as per standard procedure. Briefly, purified reduced/non-reduced protein was run on 12% SDS-PAGE followed by transfer to NC membrane and blocking with 5% skimmed milk in PBS at 37°C for 2hrs. These blots were further incubated with respective monoclonal/polyclonal antibodies diluted in 1% skimmed milk in PBS (diluent) for 1hr at 37°C followed by HRP conjugated secondary antibody (anti-mouse or anti-rabbit) for 1hr. The immunoblots were developed using 1mg/ml 3,3'-diaminobenzidine tetrahydrochloride (Sigma) and 1μl/ml H_2_O_2_ in PBS.

For detection of native parasite proteins by anti-AMSP-Fu_35_ sera, schizont stage parasite lysate was prepared from synchronized parasite culture. The culture pellet was treated with 0.15% saponin in PBS followed by PBS wash to remove haemoglobin and RBC proteins. The parasite pellet was incubated with RIPA lysis buffer (Pierce thermo scientific) for 2hrs on ice and centrifuged at 16000g for 45mins. The parasite lysate was then used for western blotting as per standard protocol using anti-AMSP-Fu_35_, PfAARP, PfMSP-1_19_ and PfMSP-3_11_ sera. The native protein bands in the parasite lysate were detected by enhanced chemiluminescence assay using West Pico ECL kit (Pierce).

### Enzyme linked immunosorbent assay (ELISA)

All ELISA reactions were carried out in 100μl reactions. Briefly, 96-well micro-titre plates (Nunc) were coated with respective antigens (200ng per well) in 0.06 M carbonate-bicarbonate buffer (pH 9.6) and incubated overnight at 4°C. Antigen coated plates were washed thrice with PBS-T and blocked with 2% skimmed milk in PBS (pH 7.2) for 2hrs at 37°C. Antigen coated plates were sequentially incubated with serial dilutions of the immune sera from mice or rabbits and then with horse radish peroxidase (HRP) labelled secondary antibodies (anti-mouse or anti rabbit IgG) for 1hr each at 37°C. The enzyme reaction was developed by a mixture of o-phenylenediamine dihydrochloride (OPD) and hydrogen peroxide (H_2_O_2_) in citrate phosphate buffer (pH 5.0); the reaction was stopped by 2N H_2_SO_4_ and OD was recorded at 492nm by Versamax ELISA reader (Molecular Devices). Cut-off values were determined as the mean plus three SDs for the pre-immunization sera and were used to determine the end point titres.

To coat the reduced protein, DTT was added to the protein sample to a final concentration of 20% (v/v), sample was incubated in boiling water for 5min and then coated onto the 96 well ELISA plate at a concentration of 200ng per well. The conformational integrity of PfMSP-1_19_ in AMSP-Fu_35_ was detected by ELISA of reduced and non-reduced protein with conformation specific monoclonal antibodies 2E10 and 1H4 [35] as described above. The monoclonal antibodies were used at a dilution of 1:1000 in triplicate wells followed by HRP-conjugated goat anti-mouse IgG (Sigma Aldrich) at a dilution of 1:1000. Also, the protein in these conditions was probed with polyclonal sera raised against the fusion construct and all the individual components.

### Immunofluorescence assay (IFA) for PfAMSP-Fu_35_

Anti-AMSP-Fu_35_ antibodies from mice and rabbits were tested for their abilities to recognize the native proteins within the parasite by immunofluorescence assays (IFA). Synchronized *P*. *falciparum* 3D7 schizont stage smears were air-dried and fixed with pre-chilled methanol at -20°C for 30 min. The fixed smears were blocked by 3% bovine serum albumin (BSA Fraction V; Sigma) in PBS pH 7.4 for 2hrs at room temperature and then washed twice for 5min each with PBS. Slides were co-immunostained with AMSP-Fu_35_ sera in combination with sera of individual components *viz* PfAARP, PfMSP-3_11_ or PfMSP-1_19_. All the sera were used at a dilution of 1:100 except PfMSP-3_11_ sera which was used at a dilution of 1:50 in 1% BSA in PBS-T (diluent) for 1hr. After washing thrice with PBS, the slides were incubated further with anti-mouse and anti-rabbit IgG conjugated to Alexa 488 and Alexa 594 respectively at 1:300 in diluent for 1hr at room temperature. Slides were subsequently washed, air dried and mounted with DAPI antifade (Invitrogen). Fluorescence and co-localization was examined under a Nikon SE300 confocal microscope with 100 X oil immersion objective.

### IgG purification and Invasion inhibition assay

Total IgGs were purified from pre-immunized (Day 0) and immunized serum (Day 70) of protein immunized rabbits using a Protein G-Sepharose column (GE Healthcare). Briefly, serum sample, after 1:1 dilution in binding buffer (20mM sodium phosphate buffer pH 7.0), was loaded onto a Protein G-Sepharose column pre-equilibrated with five column volumes of binding buffer. The column was washed with 10 column volumes of binding buffer and bound IgGs were eluted with 0.2M glycine-HCl (pH 3.0) and neutralized with 1M Tris pH 8.5. Eluted fractions were analyzed by SDS-PAGE and fractions containing purified IgGs were pooled, concentrated, dialyzed against incomplete RPMI and estimated by BCA and OD at 280nm.

Purified IgGs were tested for their ability to inhibit invasion of the parasite, *P*. *falciparum* 3D7 *in vitro*. Parasite culture was maintained and synchronized as previously described by Trager and Jenson [36]. For all the experiments, human RBCs were procured from Rotary blood bank, New Delhi, India. Synchronized parasite cultures were adjusted to 2% hematocrit and approximately 0.3% parasitaemia in late-trophozoites/early-schizonts stage. Purified IgGs (pre-immune and immune) were added to the parasite culture at final concentrations of 1, 2.5 and 5mg/ml followed by incubation for 44hr under mixed gas environment (5% O_2_, 5% CO_2_, and 90% N_2_). The efficiency of invasion inhibition was determined by scoring the percentage of infected erythrocytes by ethidium bromide (Invitrogen Corporation) staining at a concentration of 10μg/ml and acquiring the stained cells using a FACS Calibur cytometer (BD Bioscience). The percentage of infected erythrocytes was evaluated using CellQuest software (BD Bioscience) by determining the proportion of ethidium bromide-positive cells. The inhibition rate was determined according to the following: % inhibition = [(1-(Parasitemia of test IgG-Parasitemia of starting culture)/ (Parasitemia of pre-immune IgG-Parasitemia of starting culture)] × 100%.

For reversal assay, total IgGs from AMSP-Fu_35_ were pre-incubated with three different concentrations (25μg/ml, 50μg/ml, 100μg/ml) of recombinant AMSP-Fu_35_, PfMSP-1_19_, PfMSP-3_11_ and PfAARP for 1hr at 37°C following which the assay was performed as described above.

### Antibody-dependent cell inhibition (ADCI assay)

The antibody-dependent cell inhibition (ADCI) assay was performed with purified IgGs from AMSP-Fu_35_ rabbit sera according to procedure described earlier [37]. For this assay, THP1 monocyte culture stocks were obtained from American Type Culture Collection (ATCC) and maintained in complete RPMI medium containing 10% Fetal Bovine Serum (HIMEDIA). Briefly, tightly synchronized *P*. *falciparum* 3D7 schizont-stage parasites (0.5% parasitemia and 2% hematocrit) were co-cultured in a 96-well flat-bottom micro-culture plate with human monocytes (2×10^6^ monocytes per ml) in complete medium (RPMI medium containing 0.5% albumax). Purified antibodies at a concentration of 50μg/ml were added to these cultures and incubated at 37°C for 96hr in mixed-gas environment. After 48 and 72hrs of growth, 50μL of complete medium was added to each well. After 96hrs of growth, parasitemia was estimated by a two colour staining with ethidium bromide and anti-HLA-ABC antibodies and acquiring the stained cells using a FACS Calibur cytometer (BD Bioscience). Control wells consisted of (i) parasite alone, (ii) parasite and control IgG (purified from naïve rabbit sera), (iii) parasites and monocytes, (iv) parasite and purified IgG without monocytes, and (v) parasites, control IgG, and monocytes. The specific growth inhibition index (SGI) was calculated as follows: 1− [(percent parasitemia with monocytes and test antibodies/percent parasitemia with test antibodies)/ (percent parasitemia with monocytes and control IgG/percent parasitemia with control IgG)] × 100.

For reversal assay, total IgGs from AMSP-Fu_35_ were pre-incubated with three different concentrations (25μg/ml, 50μg/ml, 100μg/ml) of recombinant AMSP-Fu_35_, PfMSP-1_19_, PfMSP-3_11_ and PfAARP for 1hr at 37°C following which the assay was performed as described above.

### Statistical analysis

All data are presented as the means ± standard deviation and results were statistically analyzed. Data analyses were performed using GraphPad Prism (GraphPad software, Inc., La Jolla, CA). A *P*-value <0.05 was considered to be statistically significant.

## Results & Discussion

### Expression, purification and characterization of recombinant protein

**Fig 1:**
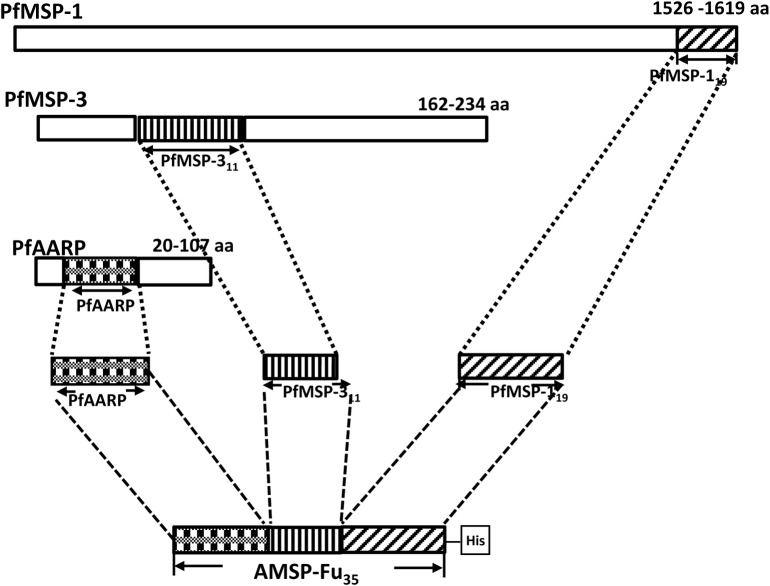
Schematic diagram of the AMSP-Fu_35_ gene consisting of PfAARP, PfMSP-3_11_ and PfMSP-1_19_. The shaded boxes for all the three proteins and the numbers on the top right corners represent the regions and the amino acid numbers of the regions which are used for the construction of AMSP-Fu_35_.

**Fig 2:**
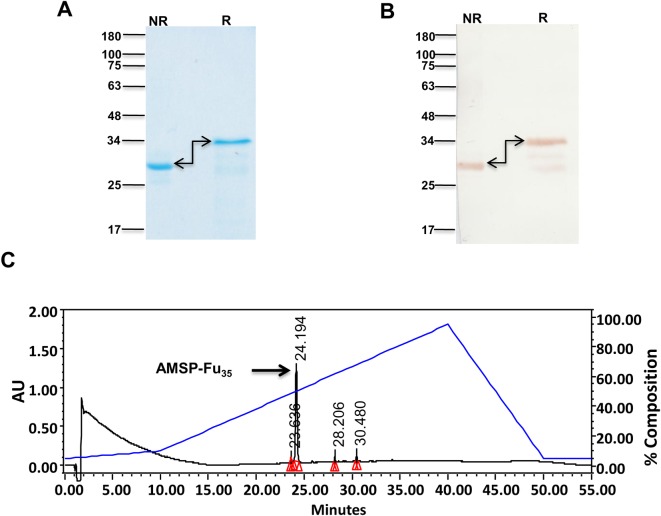
Characterization of recombinant AMSP-Fu_35_. (A) Mobility Shift of AMSP-Fu_**35**_ under reducing (R) and non-reducing (NR) conditions on SDS-PAGE. (B) Detection of AMSP-Fu_**35**_ with anti-His monoclonal antibody under reducing (R) and non-reducing (NR) conditions. (C) RP-HPLC profile of purified recombinant AMSP-Fu_**35**_ which eluted as a single peak indicating 98% purity.

Sequences of PfAARP, PfMSP-3_11_ and PfMSP-1_19_ were genetically coupled from the N to C-terminus to generate *AMSP-Fu*_*35*_, *a chimeric gene construct* with a C-terminal His tag, which was cloned in pET24b (+) (Fig 1). AMSP-Fu_35_ was expressed as a soluble protein in cytosolic fractions and purified to homogeneity by a two step chromatographic procedure. The purified protein showed an apparent mobility at ~35kDa under reducing and ~28kDa under non-reducing conditions on SDS-PAGE (Fig 2A). This shift in mobility under non-reducing conditions indicates the presence of intramolecular disulphide bonds since the construct is comprised of twelve cysteines. That all the twelve cysteine residues were engaged in disulphide bond formation was confirmed by negative Ellman test results (data not shown). Identity of the recombinant protein was confirmed by western blot analysis under reducing and non-reducing conditions with anti-His monoclonal antibody (Fig 2B). SDS-PAGE analysis also showed that the protein was purified to homogeneity. The purity of the protein was further confirmed by RP-HPLC which revealed the presence of a single symmetric peak with 98% area under the curve as calculated by the RP-HPLC profile (Fig 2C). Assessment of the endotoxin levels by using a gel clot assay based on Limulus Amoebocyte lysate (LAL) reagent kit showed that in AMSP-Fu_35_, the endotoxin level was 8 EU per 25μg of the protein which is way below the permissible limit for immunization. We were able to purify ~ 40mg AMSP-Fu_35_ of the above described quality from a 6 L shake flask culture preparation.

**Fig 3:**
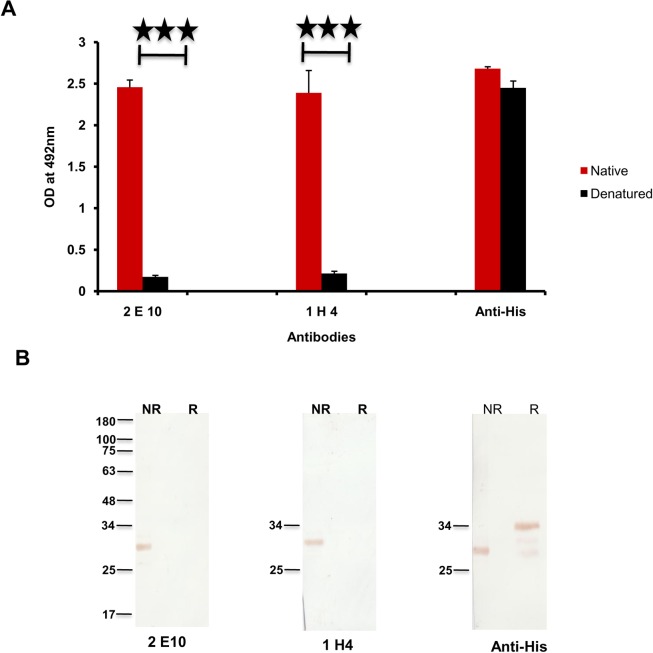
Conformational integrity of PFMSP-1_19_ in AMSP-Fu_35._ (A) Reactivity of AMSP-Fu_35_ under reducing (R) and non-reducing (NR) conditions with PfMSP-1_19_ conformational specific MAbs (2E10 and 1H4) and anti-His antibody by ELISA at a dilution of 1:1000. (B) Recognition of reduced (R) and non-reduced (NR) AMSP-Fu_35_ by PfMSP-1_19_ conformation specific MAb’s and anti-His antibody by immunoblotting.

An important criterion of successful construction of a fusion chimera is the maintenance of conformational integrity of immunologically relevant regions of the individual components, particularly of the cysteine rich regions. Since maintenance of conformational integrity of PfMSP-1_19_ is essential for its ability to inhibit parasite invasion [38], we analysed the same for AMSP-Fu_35_ using conformation specific ELISA and western blotting. Our results from both ELISA and western blotting showed that purified AMSP-Fu_35_ was strongly recognized by PfMSP-1_19_ specific MAb’s 1H4 and 2E10 confirming that proper conformation of the epidermal growth factor like domains of PfMSP-1_19_ was retained in AMSP-Fu_35_ (Fig 3A and 3B). Further, reactivity of AMSP-Fu_35_ with 2E10 and 1H4 declined to insignificant value in ELISA (OD_492_ = 0.2 for 2E10 and 0.3 for 1H4) carried out under reducing conditions, suggesting that critical epitopes of PfMSP-1_19_ in the fusion protein were present in the same conformation as in native PfMSP-1_19_. This was further confirmed by immunoblots where AMSP-Fu_35_ was recognized by the monoclonal antibodies under non-reducing conditions but was not recognized under reducing conditions (Fig 3B). Similar reactivity of AMSP-Fu_35_ with anti-His antibody under reducing and non-reducing conditions was observed in the two assays. This ascertained equal coating in ELISA and equal loading in immunoblots of reduced and non-reduced AMSP-Fu_35_. It is noteworthy that C-terminal 19kDa (PfMSP-1_19_) in this fusion and/ or other bivalent fusion proteins in *E*. *coli* is expressed as a soluble protein and that the 19kDa fragment is always appropriately folded through six disulphide bonds [26,27]. This may point out an inherent conformational preference that this EGF like domain has and may well suggest as some crucial functional role for this region of PfMSP-1 [39].

Three points are noteworthy about the production of AMSP-Fu_35._ First, the protein was expressed and purified with ease in relatively higher yields than its individual components; PfMSP-3_11_ was purified from inclusion bodies and yields of soluble PfMSP-1_19_ were low [27,33]. Second, the conformational integrity of PfMSP-1_19_ fragment was maintained which is essential for producing invasion inhibitory antibodies to this fragment. Third, ease of preparation and purification of AMSP-Fu_35_ will allow scale up of the production, if needed.

### AMSP-Fu_35_ chimera protein induced high titre antibody responses with Freund’s and Alhydrogel formulations

**Fig 4:**
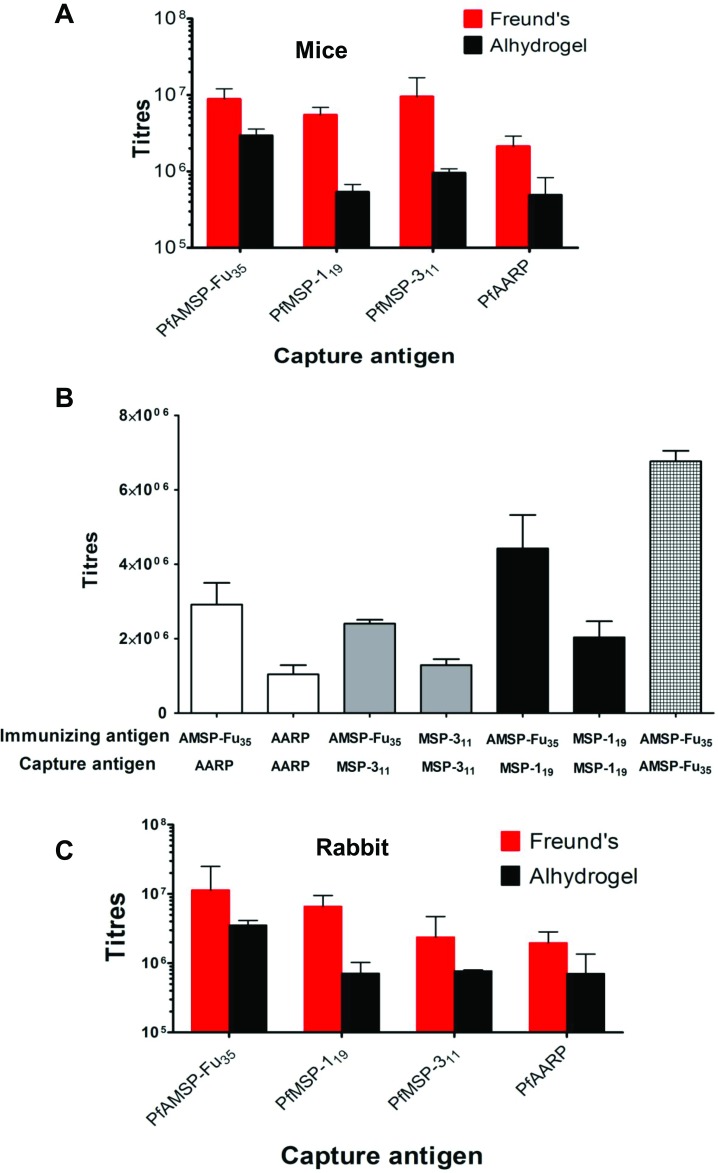
Antibody responses against AMSP-Fu_35_. (A) The end point titres against AMSP-Fu_**35**_, PfMSP-1_**19**_, PfMSP-3_**11**_ and PfAARP were measured by ELISA for Freund’s and Alhydrogel formulated AMSP-Fu_**35**_ in Balb/c mice (n = 6) (B) Comparison of the antibody response to PfMSP-1_**19**_, PfMSP-3_**11**_ and PfAARP in mice immunized with AMSP-Fu_**35**_ to the mice immunized with individual components alone (n = 5) (C) The end point titres against AMSP-Fu_**35**_, PfMSP-1_**19**_, PfMSP-3_**11**_ and PfAARP were measured by ELISA for Freund’s and Alhydrogel formulated AMSP-Fu_**35**_ in New Zealand White rabbits (n = 2).

Different sets of mice were immunized with AMSP-Fu_35_ formulated with Freund’s adjuvant and Alhydrogel. Sera from mice immunized with AMSP-Fu_35_ were tested for reactivity with AMSP-Fu_35_ itself as well as with its individual component fragments. End point titres against AMSP-Fu_35_ were highest as expected but high antibody titre were also seen against all the three constituent components, PfAARP, PfMSP-3_11_ and PfMSP-1_19_, with both the adjuvant formulations (Fig 4A). Although there was no significant difference in antibody titres to individual components, higher titres were observed for PfMSP-3_11_ followed by PfMSP-1_19_ and PfAARP (Fig 4A). In order to investigate specificity of antibody response to the three components and also to compare the antibody response induced by AMSP-Fu_35_ immunization to that by immunization with individual components, mice were also immunized with PfAARP, PfMSP-3_11_ and PfMSP-1_19_, formulated separately with Freund’s adjuvant (Fig 4B). Immunization with the three component antigens also produced reasonable antibody response but results of ELISA showed that not only high antibody titres were induced to the individual components but also the response was significantly higher to all the three components (p = 0.0115, 0.0004 and 0.0469 for PfAARP, PfMSP-3_11_ and PfMSP-1_19_ respectively) in case of immunization with AMSP-Fu_35_.

Immunization with AMSP-Fu_35_ in rabbits formulated in Freund’s adjuvant also produced high antibody titres to AMSP-Fu_35_ (~ 10^7^) as well as to the three individual components (10^6^) (Fig 4C). The end point titres with Alhydrogel formulations were of the order ~10^6^ to AMSP-Fu_35_ and ~10^5^ to the individual components.

The above results also indicated that there was no antigenic interference upon fusion of the three antigen fragments and that each component remained immunologically functional with regard to antibody response. However, lack of such an antigenic competition has been reported earlier for other malaria fusion proteins [23,24,27,33]. These results highlight the potential of the approach of using fusion chimeras comprised of key antigenic determinants of different antigens for subunit vaccine development.

### Anti-AMSP-Fu_35_ antibodies recognize native proteins of *P*. *falciparum*

**Fig 5:**
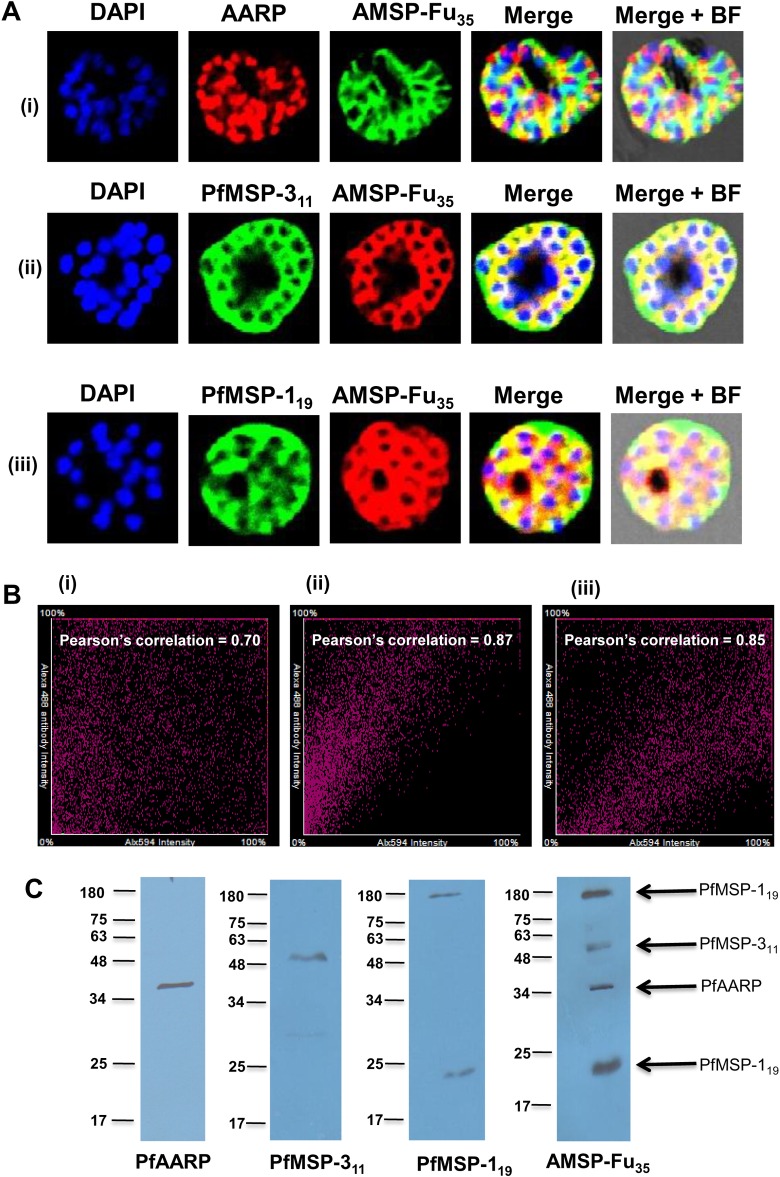
Native conformations of the three individual components in AMSP-Fu_35_. (A) IFA was performed with late schizont stage parasites using AMSP-Fu_**35**_ sera and co-localization was checked by co-immunostaining with sera against individual components (anti-PfMSP-1_**19**_, PfAARP and PfMSP-3_**11**_). The corresponding images stained with DAPI, which stains nuclear DNA; merged fluorescence images and bright field images are also shown. (B) Pearson’s correlation graphs for the co-immunostained slides, (i) AMSP-Fu_**35**_ and PfAARP, (ii) AMSP-Fu_**35**_ and PfMSP-3_**11**_ and (iii) AMSP-Fu_**35**_ and PfMSP-1_**19**_, are shown and high correlation values showed co-localization. (C) Antibodies to AMSP-Fu_**35**_ react with proteins corresponding to PfAARP, PfMSP-1_**19**_ and PfMSP-3_**11**_ in immunoblots of schizont stage parasite lysate from 3D7. Parallel immunoblots of the same lysate probed with anti-AMSP-Fu_**35**_, PfAARP, PfMSP-3_**11**_ and PfMSP-1_**19**_ sera confirmed the proteins detected by the fusion sera.

Immune sera that were raised against AMSP-Fu_35_ in mice and rabbits using two different adjuvants were assessed for their ability to recognize the native parasite proteins expressed during the blood stages of the parasite by immunofluorescence (IFA) and immunoblotting of schizont stage parasite lysate (Fig 5A, 5B and 5C). Sera against the fusion chimera showed strong reactivity on surface of *P*. *falciparum* schizonts indicating the recognition of native parasite proteins by fusion antisera (Fig 5A). High values of Pearson’s correlation were observed on co-immunostaining of parasite with sera against the fusion chimera and that of the individual components *viz* anti-PfAMSP-Fu_35_ sera and separately with anti-PfAARP, PfMSP-3_11_ or PfMSP-1_19_ (Fig 5B). These results showed that proteins detected by anti-PfAMSP-Fu_35_ antibodies co-localized with those detected by sera against individual components. However, owing to the inherent limitations with IFA like cross reactivity which may result in false positives, recognition of native parasite proteins was further confirmed by immunoblotting schizont stage parasite lysate with anti-AMSP-Fu_35_ sera which detected a number of bands, at ~ 19kDa, ~35kDa, ~ 47kDa and ~190kDa (Fig 5C). When the same lysate was probed with the sera against the individual components, we observed that anti-PfAARP sera recognized a ~35kDa protein, anti-PfMSP-3_11_ sera recognized a protein at ~ 47kDa and anti-PfMSP-1_19_ sera recognized two bands, one at ~ 19kDa and other at ~ 190 kDa. Thus, these results clearly showed that anti-AMSP-Fu_35_ sera recognized native proteins in the parasite lysate corresponding to the three individual components of the fusion chimera.

### *In vitro* parasite invasion inhibition by anti-AMSP-Fu_35_ antibodies

**Fig 6:**
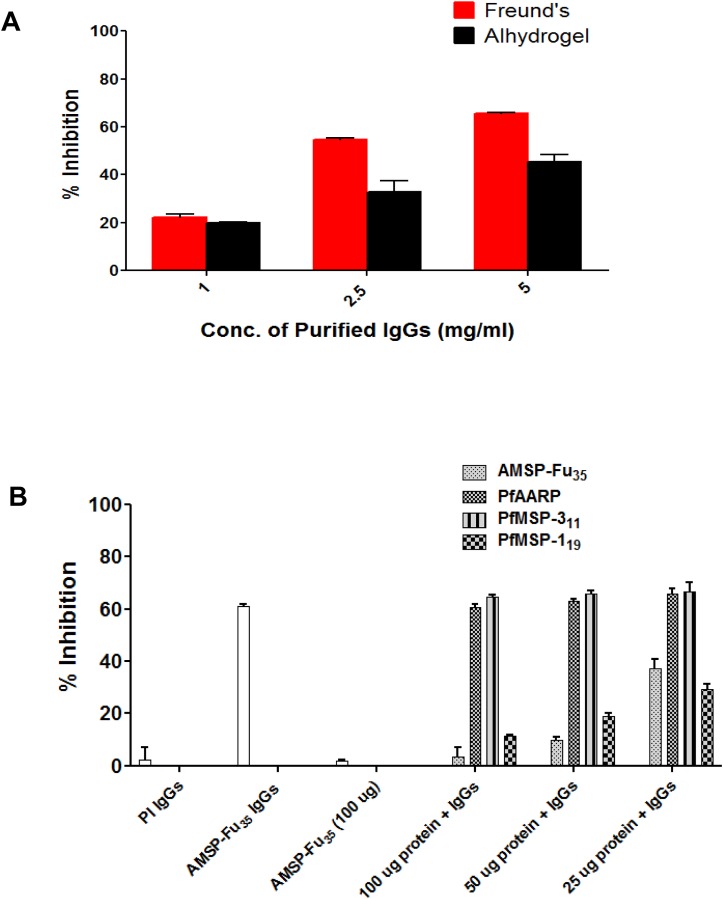
Inhibition of parasite invasion *in vitro* by antibodies specific to AMSP-Fu_35_ and specificity of the inhibition observed. (A) IgGs purified from rabbit sera immunized with AMSP-Fu_**35**_ formulated with different adjuvants were tested at 1mg/ml, 2.5mg/ml and 5mg/ml for inhibition of erythrocyte invasion in *P*. *falciparum* 3D7. Percent growth inhibition (shown as means ± standard deviations) was calculated by using the parasitemia in the presence of immune sera with respect to parasitemia in the presence of pre-immune sera. (B) Purified IgGs against AMSP-Fu_**35**_ was tested for its invasion inhibitory specificity against the 3D7 parasite clone. For studying the invasion inhibitory specificity, total IgGs were pre-incubated with recombinant proteins AMSP-Fu_**35**_, PfMSP-1_**19**_, PfMSP-3_**11**_ and PfAARP, at the final concentrations of 25μg/ml, 50μg/ml and 100μg/ml. The white bar denotes the invasion inhibitory activity of AMSP-Fu_**35**_ IgGs.

IgGs purified from rabbits immunized with AMSP-Fu_35_ formulated with Freund’s and Alhydrogel adjuvants were tested for their ability to inhibit *in vitro* parasite invasion of *P*. *falciparum*. Results showed that total IgGs from both adjuvant formulations inhibited parasite invasion in a concentration dependent manner in the range from 1.0mg/ml to 5.0mg/ml of total IgGs; there was up to 65% and 40% inhibition in Freund’s and Alhydrogel formulations respectively, at a concentration of 5mg/ml (Fig 6A).

To study fine specificity of the observed invasion inhibition, antibodies specific to AMSP-Fu_35_, PfAARP, PfMSP-1_19_, or PfMSP-3_11_ were depleted from purified anti-AMSP-Fu_35_ IgGs by incubation with different concentrations (25μg/ml, 50μg/ml and 100μg/ml) of recombinant AMSP-Fu_35_, PfAARP, PfMSP-1_19_, and PfMSP-3_11_, respectively. These antibody samples depleted of specific IgGs were then assessed for their ability to inhibit invasion of the parasite (Fig 6B). Our results showed dose dependence in reversal of parasite invasion inhibition and at a concentration of 100μg/ml of AMSP-Fu_35_ inhibition reversal was ~ 95% whereas with PfMSP-1_19_ it was about 80% at the same concentration of PfMSP-1_19_. Antibody depletion with PfAARP or PfMSP-3_11_ did not alter invasion inhibition suggesting that the antibody component specific to these two proteins do not significantly contribute to observed invasion inhibition. While anti-PfMSP-3_11_ antibodies do not directly inhibit parasite invasion but instead are known to kill parasite in monocyte dependent manner, anti-PfAARP antibodies have been shown to inhibit parasite invasion effectively alone and in combination with antibodies to other parasite antigens [21,31]. That depletion of anti-PfAARP antibodies had no significant effect on parasite inhibition was surprising. It may be that anti-PfMSP-1_19_ antibodies in these experiments are alone so efficient in inhibition of parasite invasion that additional synergy from anti-PfAARP antibodies is not observed. In fact, the rationale for the present study was to explore whether addition of AARP to the two components based MSP-Fu_24_ would enhance the antigenicity of AMSP-Fu_35_ and whether the triple construct will be a better vaccine candidate than MSP-Fu_24_. Our results of antigenicity and other experiments showed that although, unfortunately, addition of AARP to MSP-Fu_24_ did not provide synergistic effects in invasion inhibitory activity, these results have shown that more than two immunologically relevant regions may successfully be combined in a single construct without any immunological interference. However, immunogenicity of any such constructs will need to be tested case by case. These results have also shown that AMSP-Fu_35_ is as good an immunogen as MSP-Fu_24_ in *in vitro* assays.

### Anti-AMSP-Fu_35_ antibodies inhibit parasite growth in an ADCI assay

**Fig 7:**
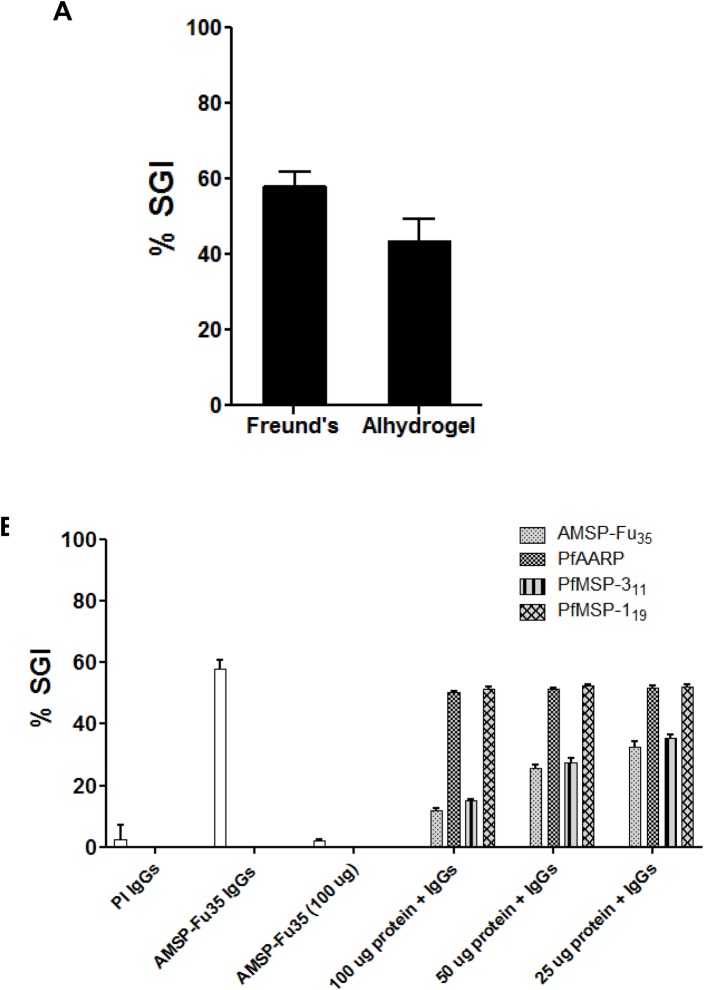
Monocyte mediated inhibition of parasite invasion *in vitro* by antibodies specific to AMSP-Fu_35_ and the three individual components. (A) *P*. *falciparum* 3D7 parasites from the schizont stages were co-cultured with human monocytes in the presence of 50**μ**g of purified IgGs from rabbits immunized with AMSP-Fu_**35**_ formulated with Freund’s adjuvant and Alhydrogel. Parasite growth and multiplicity was analyzed after 96hrs. SGI index for ADCI of parasite growth (shown as means ± standard deviations) was calculated by using the parasitemia of test IgGs in the presence and absence of monocytes with parasitemia of control IgGs in the presence and absence of monocytes. (B) Purified IgGs against AMSP-Fu_**35**_ was tested for its invasion inhibitory specificity against the 3D7 parasite clone. For studying the invasion inhibitory specificity, total IgGs were pre-incubated with recombinant proteins AMSP-Fu_**35**_, PfMSP-1_**19**_, PfMSP-3_**11**_ and PfAARP, at the final concentrations of 25μg/ml, 50μg/ml and 100μg/ml. The white bar denotes the invasion inhibitory activity of AMSP-Fu_**35**_ IgGs.

The present fusion chimera is based on proteins which inhibit parasite invasion both directly and in monocyte dependent manner. The fusion chimera, AMSP-Fu_35_ contained a fragment of PfMSP-3, a malaria protein, antibodies to which are well known to inhibit parasite growth by ADCI mechanism [37]. Immunization with AMSP-Fu_35_ raised antibodies specific to PfMSP-3_11_ component as detected by ELISA and since these antibodies recognized native parasite proteins as seen by IFA of schizont stage parasite and immunoblots of parasite lysate, we therefore tested whether anti-AMSP-Fu_35_ antibodies are also capable of inhibiting parasite growth by this mechanism. Results of ADCI assay showed that at a concentration of 50μg/ml, anti- AMSP-Fu_35_ IgGs showed potent ADCI effect in *P*. *falciparum* 3D7 with an SGI activity of 60% and 45% with Freund’s and Alhydrogel adjuvant formulations respectively (Fig 7A).

To further confirm that ADCI activity is indeed due to anti-MSP-3_11_ antibodies, we depleted antibodies specific to AMSP-Fu_35_, PfAARP, PfMSP-3_11_ and PfMSP-1_19_ from AMSP-Fu_35_ IgGs by incubation with different concentrations (25μg/ml, 50μg/ml and 100μg/ml) of recombinant AMSP-Fu_35_, PfAARP, PfMSP-1_19_, and PfMSP-3_11_, respectively. The depleted IgGs were used to assess the ADCI activity and, as expected, depletion of AMSP-Fu_35_ and PfMSP-3_11_ caused a dose dependence reversal in the SGI activity (Fig 7B). At a protein concentration of 100μg/ml, the reversal in SGI activity observed were about 77% for AMSP-Fu_35_ and about 67% for PfMSP-3_11_. However, no reversal was observed on depletion of PfAARP or PfMSP-1_19_ specific antibodies. These results provide a more direct evidence for the generation of functional antibodies to MSP-3_11_ fragment upon AMSP-Fu_35_ immunization. From the results of these two independent invasion inhibition assays (direct and monocyte dependent), it is quite clear that both PfMSP-1_19_ and PfMSP-3_11_ were functionally expressed in the fusion chimera.

## Conclusion

Here we have described design, production, biochemical and antigenic characterization of a fusion protein comprised of three prominent blood stage malaria vaccine target antigens. The recombinant fusion protein is easy to produce and purify to homogeneity. Immunization with the fusion chimera induced high titre antibodies to the individual components without any antigenic competition. Antibodies against AMSP-Fu_35_ recognize native proteins corresponding to its three components in the schizont stage parasites. These antibodies inhibited parasite growth *in vitro* efficiently by invasion inhibitory and ADCI mechanisms.

The rationale for designing of AMSP-Fu_35_ containing an additional immunodominant region from PfAARP was to develop a construct which would exhibit enhanced immunogenicity vis-à-vis invasion inhibitory activity than the previously described two components based MSP-Fu_24_. Although AMSP-Fu_35_ was highly immunogenic, it did not provide any observable enhancement to the invasion inhibitory activity of MSP-Fu_24_. Given that, MSP-Fu_24_ is highly immunogenic and also easy to produce in high yields than AMSP-Fu_35_, it may not be of much merit to consider AMSP-Fu_35_ as a vaccine candidate for pre-clinical development. Development of MSP-Fu_24_ for pre-clinical evaluation is under progress in our laboratory.
